# Platelet and Fibrinogen Contribution to Clot Strength in Premature Neonates with Sepsis

**DOI:** 10.3390/children12070948

**Published:** 2025-07-18

**Authors:** Dimitra Gialamprinou, Christos-Georgios Kontovazainitis, Abraham Pouliakis, Alexandra Fleva, Anastasia Giannakou, Elisavet Diamanti, Panagiotis Kratimenos, Georgios Mitsiakos

**Affiliations:** 1 2nd Neonatal Department and Neonatal Intensive Care Unit (NICU), Aristotle University of Thessaloniki, “Papageorgiou” University Hospital, Ring Road, Municipality of Pavlou Mela, Area N. Evkarpia, 56403 Thessaloniki, Greece; gialamprinou@gmail.com (D.G.); ckontova@auth.gr (C.-G.K.); diamantie@auth.gr (E.D.); 2 2nd Department of Pathology, National and Kapodistrian University of Athens, “Attikon” University Hospital, Rimini 1, Area Chaidari, 12462 Athens, Greece; apouliak@med.uoa.gr; 3 Immunology and Histocompatibility Department, “Papageorgiou” University Hospital, Ring Road, Municipality of Pavlou Mela, Area N. Evkarpia, 56403 Thessaloniki, Greece; alexfleva@gmail.com (A.F.); giannakouan@gmail.com (A.G.); 4 Division of Neonatology, Children’s National Medical Center, 111 Michigan Avenue NW, Washington, DC 20010, USA; pkratimen2@childrensnational.org; 5 School of Medicine and Health Sciences, George Washington University, Ross Hall, 2300 Eye Street NW, Washington, DC 20037, USA

**Keywords:** neonatal sepsis, thromboelastometry, platelet function tests, thrombocytopenia, fibrinogen, platelet transfusion

## Abstract

**Background/Objectives**: Platelet transfusions are administered to preterm neonates with thrombocytopenia prophylactically to decrease their bleeding risk. The amplitude difference between the extrinsic rotational thromboelastometry (EXTEM) and the fibrinogen rotational thromboelastometry (FIBTEM) assays is considered an index of platelet contribution to clot strength, guiding transfusion management. The difference in maximum clot elasticity (MCE) (namely the platelet contribution to clot elasticity—MCEplatelet) is considered highly accurate. Limited data exist to specify the contribution of platelets and fibrinogen in clot formation during sepsis in neonates with thrombocytopenia. We investigated the potential of MCFplatelet (platelet contribution to clot firmness) and MCEplatelet in reflecting platelet count and function in septic preterm neonates. We simultaneously assessed the contribution of both platelets and fibrinogen to clot strength during sepsis. **Methods**: We compared 28 preterm neonates with sepsis born (gestational age 24^+1^-34^+3^) with 30 healthy counterparts by using rotational thromboelastometry (ROTEM) and platelet flow cytometry. **Results**: MCEplatelet showed a higher association with platelet count in the sepsis group than MCFplatelet (R^2^ = 47.66% vs. R^2^ = 18.79%). MCEplatelet (AUC = 0.81) had better discrimination capability than MCFplatelet (AUC = 0.78) in platelet count <100 × 10^3^/L. MCEplatelet was poorly associated with platelet function. The contribution of platelets was significantly lower (MCEplatelet = 84.03 vs. 89.21; *p* < 0.001) compared with fibrinogen (36.9 vs. 25.92; *p* < 0.001) in the sepsis group. **Conclusions**: MCEplatelet has a better predictive value than MCFplatelet. In clinical practice, the elasticity difference between EXTEM and FIBTEM may replace the amplitude difference. The higher contribution of fibrinogen in clot strength during neonatal sepsis results in higher MCF, even in neonates with thrombocytopenia.

## 1. Introduction

Thrombocytopenia is a common complication among septic neonates [1]. The incidence of thrombocytopenia in neonates with sepsis is approximately 40% with severe thrombocytopenia, namely a platelet count below 50,000/μL, accounting for a percentage of 20%, depending on the study. Severe thrombocytopenia has been associated with major bleeding and the overall mortality in neonatal sepsis, reaching a percentage up to 10%, although a clear causative relation between low platelet counts and bleeding risk has not been established [1]. Prophylactic platelet transfusion is considered to reduce the risk of bleeding in preterm sick thrombocytopenic neonates. Variable transfusion strategies have been recorded among institutes. American neonatologists administer platelet transfusions at higher platelet counts than their counterparts in the UK, with thresholds of ~70,000/μL and 27,000/μL, respectively. The results of the Platelets for Neonatal Transfusion-study 2 (PlaNeT-2) demonstrated that neonates with the highest baseline risk for bleeding or mortality according to the gestational age and clinical status benefited from transfusions based on a restrictive threshold of platelets 25,000 PLTs/μL [2]. Guidelines from the British Society of Hematology for platelet transfusion in neonates suggest that for preterm neonates or non-bleeding neonates, platelet transfusions should not be administered if the platelet count is above 25,000/μL, irrespective of the clinical conditions, including sepsis [3]. Additionally, the recent literature highlights the harmful effects of platelet transfusions in premature neonates regarding neurodevelopmental impairment, while the indicative platelet count for transfusion varies among neonatal groups [4]. Viscoelastic tests have been widely used in adults for restricting redundant transfusions. Still, it is questionable if they may be applied in Neonatal Intensive Care Units (NICUs) [5,6].

The clot amplitude derived from viscoelastic tests is currently assessed as an index of clot strength. Algorithms based on rotational thromboelastometry (ROTEM) assays for bleeding treatment suggest a low clot amplitude at 10 min < 40 mm (A10 < 40 mm) in the Extrinsic ROTEM (EXTEM) assay as a trigger for transfusing platelets [7,8]. Since the platelet component of clot strength may be more indicative of platelet deficiency than clot amplitude, it has been recently suggested that platelet contribution to clot strength may be accurately provided by using the difference in maximum clot elasticity (MCE) rather than maximum clot firmness (MCF) between the EXTEM and Fibrinogen ROTEM (FIBTEM) assay [9,10]. Furthermore, clot amplitude represents the overall contribution of red blood cells, platelets, and fibrinogen in clot strength [11].

In neonatal sepsis, the innate immune proinflammatory response leads to hypercoagulation in terms of “immunothrombosis,” [12,13] and fibrinogen dominates blood coagulability [14]. Studies in thrombocytopenic patients with inflammatory diseases have shown that increased fibrinogen levels could compensate for a low platelet count, reducing the risk of bleeding [15,16]. Studies in neonates have shown a very poor correlation between the severity of thrombocytopenia and the risk of clinically significant bleeding [17]. The determinant role of fibrinogen may be considered for decision-making regarding transfusion strategy in thrombocytopenic neonates.

So far, the potential of platelet elasticity to better provide platelet count and function has been scarcely investigated [18]. In neonates, the correlation between platelet clot amplitude or platelet clot elasticity and platelet count and function remains to be elucidated. Furthermore, the contribution of both platelets and fibrinogen in clot strength should be clarified, as a simple difference in amplitude between EXTEM and FIBTEM may be a misleading index for platelet transfusions in neonates with sepsis. Thus, this study aimed to investigate the ability of platelet contribution to clot elasticity (MCEplatelet) and platelet contribution to clot firmness (MCFplatelet) to reflect platelet count and function, simultaneously assessing the role of platelets and fibrinogen in clot strength during neonatal sepsis. We hypothesized that ROTEM-derived parameters, such as MCEplatelet, would accurately reflect platelet function and count in septic preterm neonates.

## 2. Materials and Methods

The study was conducted under a protocol number (05.02.2019/3696) in accordance with the Declaration of Helsinki and approved by the Institutional Review Board of Papageorgiou General Hospital of Thessaloniki. All blood samples of neonates were taken after the parents’ written informed consent.

### 2.1. Study Design and Population

This prospective cohort study enrolled preterm neonates with confirmed sepsis hospitalized in the NICU of a tertiary general hospital over a period of 25 months (October 2019–October 2021). These patients were compared with healthy preterm neonates, who served as controls. The detailed methodology is described in a previously published report [12]. Prematurity was defined as birth with gestational age below 37 weeks. The last ultrasound during the first trimester of pregnancy, and always before 13 6/7 gestational weeks, was used for calculating the gestational age. The inclusion criteria for the study were adapted from “The Third International Consensus Definitions for Sepsis and Septic Shock (Sepsis-3)” and the European Medicines Agency definition for neonatal early-onset sepsis or late-onset sepsis, considering the recent literature in defining neonatal sepsis [19,20,21]. The Score for Neonatal Acute Physiology Perinatal Extension II (SNAPPE II) and the Tοllner score were calculated [22,23]. Neonates with congenital anomalies, chromosomal defects, metabolic diseases, maternal thrombophilia, and familial thrombotic or bleeding medical history, as well as infants who received blood products before enrolment or who had severe perinatal asphyxia, were excluded.

The method used for matching patients with controls was based on “individual matching”. More precisely, each neonate with sepsis was matched with 1 healthy neonate of every new admission and was chosen by fulfilling the baseline characteristics initially regarding the gender and gestational age (GA), while this process was conducted during the same time frame. Specifically, the patients were matched for gestational age, gender, birth weight, day of study entry, weight on the day of study entry, and maternal and postnatal morbidity.

The populations were assessed for hemostatic and platelet function alterations during sepsis using ROTEM and platelet flow cytometry. Blood specimens for culture, routine biochemical tests, complete blood cell count, peripheral blood smear, and C-Reactive Protein (CRP) were obtained at the onset of sepsis and before initiating antibiotic therapy. Blood samples were collected at 3 time points: on the 1st, 2nd to 3rd, and 5th to 7th day of sepsis for the patient group and at the first time point for the controls. To minimize ex vivo platelet activation, blood samples were processed within approximately 30 min of the blood being drawn for both ROTEM and flow cytometry.

Blood sample for culture was obtained under aseptic techniques and was placed in an aerobic blood bottle (BACT/ALERT PF Plus, bioMerieux, Inc., Singapore). The whole blood parameters, biochemical parameters, and CRP were evaluated using Alinity hq and c analyzers, respectively (Abbott GmbH, Wiesbaden, Germany). Conventional coagulation tests (CCTs) were performed using a STA-R MAX-Stago coagulation analyzer (DIAGNOSTICA STAGO S.A.S., Asnières-sur-Seine, France) in platelet-poor plasma, obtained by centrifugation at 1750× *g* for 15 min after 500 μL of blood mixed with 0.109 mol/L trisodium citrate was obtained.

Missing data (4.1%) were deleted since they were “missing completely at random”.

### 2.2. Rotational Thromboelastometry (ROTEM)

A total of 300 μL of blood (300 μL × 4) mixed with 0.109 mol/L trisodium citrate was analyzed in a ROTEM-DELTA Analyzer (Term Innovations GmbH, Munich, Germany). Two ROTEM tests were performed for this study: the EXTEM and FIBTEM modules.

MCF was the used parameter indicating the final strength of the thrombus after an observation period following clotting time (CT). The study’s results pertain to the modifications of the referred indices MCF and MCE from the initiation and during sepsis, and in comparison, to relevant measurements from the control group. MCF data are expressed as amplitude oscillation (mm) in all ROTEM tests. MCF was properly converted to MCE (dimensionless) using a standardized formula according to the relevant literature: 100 x amplitude/(100-amplitude) and producing MCE-EXTEM and MCE-FIBTEM [9]. Depending on the above variables, platelet contribution to clot strength was calculated separately based on amplitude and based on elasticity. MCFplatelet was calculated based on the following formula: MCFplatelet = (MCF-EXTEM) − (MCF-FIBTEM). MCF is provided by ROTEM from the EXTEM and FIBTEM assays. MCE is calculated as MCE = (100 x MCF)/(100MCF) (separately for EXTEM and FIBTEM)

We assessed the contribution of fibrinogen and platelets to clot strength. The contribution of platelets in EXTEM-MCF is obtained when MCFplatelet is divided by MCF found in EXTEM. Similarly, the contribution of platelets EXTEM-MCE is obtained when MCEplatelet is divided by MCE found in EXTEM. The fibrinogen contribution in EXTEM-MCF is obtained by dividing the MCF in FIBTEM by the respective parameters in EXTEM. We investigated the determinant role of fibrinogen in clot firmness by using cut-off points in MCF-EXTEM < 55 mm, when a significant reduction in clot strength is observed. The cut-off point was selected based on the median calculation of MCF-EXTEM derived from our institution’s pool of neonatal ROTEM results between August 2018 and September 2024. A brief glossary regarding the above formulas and definitions is given in Appendix A.

### 2.3. Flow Cytometry Analysis

A total of 500 μL of whole blood was required to evaluate platelets’ function using flow cytometry by using Cytomics FC 500 (Beckman Coulter, Singapore) and managed by a biologist. A minimum of 5000 platelets was gated by their characteristic forward and side scatter, using a Flow Cytometer FC 500 (Beckman Coulter). To prepare Platelet-Rich Plasma (PRP), 500 μL of anticoagulated whole blood was centrifuged in a Centrifuge Hettich (Andreas Hettich, Tuttlingen, Germany) for 10 min at 150 to 200× *g* at room temperature. Supernatant PRP was carefully removed, and the platelet count was evaluated based on the neonate’s total blood count. In a platelet count of >150,000/μL, a dilution by modified HEPES/Tyrode’s buffer achieved a concentration of 10,000 to 15,000 platelets/μL. Based on an analytical calibration, we estimated the number of surface antigens expressed on a single platelet using a reversed cubic curve fit and quantitative beads. The results were expressed as the Mean Fluorescence Intensity (MFI) obtained from the positive platelets (% positive platelets on each surface antigen) and as an absolute number of receptors. The data were obtained using the CXP software version 1.0. A total of 100 μL of PRPs was placed into 2 Radioimmunoassay (RIA) tubes. One tube consisted of a sample of nonactivated platelets, whereas the other tube contained the activated platelets with the addition of 10 μL Thrombin Receptor-Activating Peptide (TRAP) (371 μM, JRT) as an agonist. The tube in which the TRAP was added was incubated at room temperature for 20 min. Out of these 2 tubes, we created 4 tubes for each inactivated and activated state of platelets. Antibodies against P-selectin CD62P FITC (Clone AK4) for the evaluation of α-granule expression and GPIIb (Glycoprotein IIb) CD41 FITC (Clone MEM-06), GPIb (Glycoprotein Ib) CD42b FITC (Clone AK2), and GPIIIa (Glycoprotein IIIa) CD61 FITC (Clone VIPL2) for the evaluation of the Glycoproteins (GPs) (EXBIO Praha, a.s., Vestec, Czechia) were added according to the manufacturer’s directions. The expression of antibodies against GPIb, GPIIb, GPIIIa, and P-selectin was determined in all samples by estimating the MFI and the absolute number of receptors on gated platelets [12,24]. To minimize ex vivo platelet activation, blood samples were processed within approximately 30 min of the blood draw. The act of drawing blood is itself a potential source of functional platelet activation. Therefore, we sampled arterial blood to eliminate the need for a tourniquet, and a 21-gauge butterfly needle was used to provide a smooth draw.

### 2.4. Statistical Analysis

Categorical data were presented by frequencies and percentages, and numerical data by mean or median and Standard Deviation (SD) or Interquartile Range (IQR). The nonparametric paired-sample Wilcoxon’s signed-rank test and Mann–Whitney U test were used for the analysis; the significance level was set at *p* < 0.05. A correlation analysis was performed using Spearman’s rank correlation coefficient. Correlations were considered strong if the correlation coefficient was R-Spearman ≥ 0.6 (or ≤−0.6 in case of a negative correlation), and the significance level was set at *p* < 0.05. Furthermore, MCFplatelet and MCEplatelet were tested for possible associations using linear and cubic regression models. Finally, additional factors that could affect this relation were investigated using a multivariate regression analysis, and the outcomes of this analysis were subsequently evaluated using a sensitivity analysis.

## 3. Results

**Figure 1:**
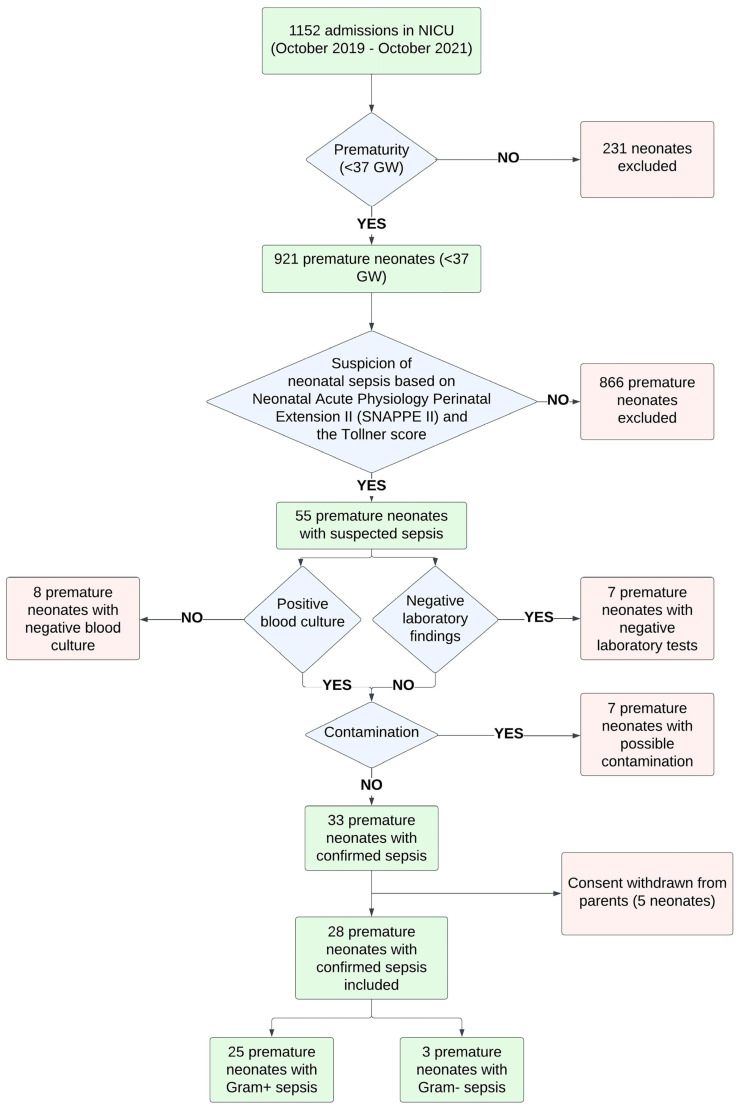
Population diagram for preterm neonates with sepsis.

Out of 1152 admissions to the NICU, 55 patients had suspected sepsis based on their clinical course and following the criteria for sepsis described in our research protocol (see below). Twenty-two patients were excluded either because of negative blood culture (N = 8) or because of suspected contamination of the blood culture and negative laboratory findings (N = 14). Out of the 33 patients with confirmed sepsis, 2 were early-onset and 31 were late-onset. Out of the 33 enrolled patients, 5 were removed because their parents decided to withdraw their consent. Finally, 28 patients were included and matched with 30 healthy counterparts, who served as the control group (Figure 1).

### 3.1. Baseline Characteristics of the Study Population

A total of 112 blood samples were collected and tested using both ROTEM and platelet flow cytometry. Out of 1152 admissions, 28 preterm neonates of a median GA of 31.4 weeks and a median birthweight of 1390 g presented with confirmed sepsis and were finally included. A total of 25 of them presented with late-onset sepsis from Gram-positive bacteria (coagulase-negative Staphylococci), and 1 had late-onset sepsis from a Gram-negative bacterium (Enterobacter cloacae), whereas 2 had early-onset sepsis from Gram-negative bacteria (*Escherichia coli*). Only neonates with sepsis from Gram-negative bacteria developed fatal DIC, severe thrombocytopenia, and severe Intraventricular Hemorrhage (IVH) of grade III and IV. Thrombocytopenia < 150,000/μL was identified in 32% of neonates with sepsis. The comparative baseline characteristics between patients and controls for the study population are depicted in Table 1. No statistical differences were recorded regarding the baseline characteristics between patients and controls (*p* > 0.05 for all tests).

### 3.2. Conventional Coagulation Tests (CCTs)

The comparative results for classical laboratory tests are presented for the sepsis and control group, with significant differences between the groups in fibrinogen, Prothrombin Time (PT), Activated Partial Prothrombin Time (aPTT), International Normalized Ratio (INR), D-dimers, protein C, White Blood Cells (WBCs), platelets, and CRP over the study period (Supplementary Table S1).

### 3.3. ROTEM Parameters

Thromboelastometry parameters for clot strength were significantly higher in the sepsis group (Table 2). In EXTEM, CT and clot formation time (CFT) were significantly lower, and A10 and MCF were higher in the sepsis group. In FIBTEM A10, MCF was significantly higher, and LI30, LI45, and LI60 were lower in the sepsis group. MCFplatelet was significantly lower in the sepsis group.

### 3.4. Flow Cytometry Parameters

The expression of GPIb was significantly higher in the platelets of neonates with sepsis than in healthy controls in both the activated and inactivated states at all time points, which is indicative of a steady increase during sepsis. The expression of GPIIb was significantly higher in the platelets of neonates with sepsis than in healthy counterparts in the inactivated states at all time points. The expression of GPIIIa was significantly increased in the platelets of patients with sepsis in the inactivated state on Day 1 and Days 5–7, compared with healthy controls. The amount of P-selectin representing platelet α-granules was significantly lower in platelets in their inactivated state from neonates with sepsis compared to healthy controls at all time points (Table 2).

### 3.5. MCFplatelet and MCEplatelet Linearity Association

**Figure 2:**
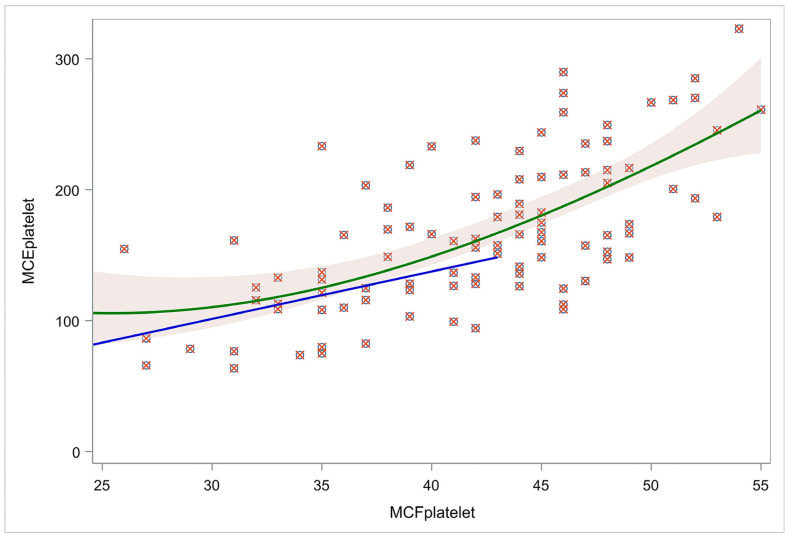
Cubic and linear models for MCFplatelet and MCEplatelet. The green line represents a cubic regression model visualizing the relation of MCEplatelet with MCFplatelet for the whole population. The blue line represents a linear regression model of the relation of MCEplatelet with MCFplatelet for those neonates with MCFplatelet < 43. Using a cut-off limit of amplitude difference (43 mm), the linear model (blue line) had R2 = 0.14, indicative of no fit between MCEplatelet and MCFplatelet, confirming their non-linear association. Abbreviations: MCFplatelet: Maximum clot firmness for platelet; MCEplatelet: Maximum clot elasticity for platelet.

MCFplatelet and MCEplatelet were tested for linear association. Both linear and cubic regression were performed to assess any association between amplitude-based platelet contribution (MCF) and the elasticity-based platelet contribution (MCE) to clot strength. According to the results, the cubic model approach (R^2^ = 0.45) had a better fit than the linear model (R^2^ = 0.40). Particularly, when using a cut-off limit of 43 mm in amplitude difference, the linear model had an R^2^ of 0.14 for those neonates that had MCFplatelet < 43 mm, indicative of no fit between MCEplatelet and MCFplatelet. Thus, the latter confirms the non-linear association (Figure 2).

Furthermore, we investigated other factors that may affect MCEplatelet values. Specifically, we performed multivariate regression to evaluate the relationship of MCEplatelet with MCFplatelet, also controlling for gestational age, maternal age, weight, gender, and platelet count. We found no correlation for MCEplatelet with GA (r = 0.1597, *p* = 0.0926), nor with maternal age (r = 0.0566, *p* = 0.6761) or gender (MCE platelet median 155.8, Q1–Q3: 121.3–180.9 for females and 152.8, Q1–Q3: 127.3–194.9 for males, *p* = 0.7675). However, a strong correlation for MCEplatelet with platelet count was found (r = 0.6517, *p* < 0.0001). Eventually, we performed a multivariable regression analysis using three factors with a *p*-value of <0.2: GA, MCFplatelet, and platelet count. GA was not a significant independent variable for MCEplatelet (estimate = −1.69; standard error = 1.24; *p* = 0.1761). In contrast, the other two factors played a significant role in MCEplatelet value estimation, i.e., estimates: 3.47 and 0.19, with standard errors: 0.58 and 0.03 for MCFplatelet and platelet count, respectively (*p* < 0.0001 for both). The model had R^2^ = 56.7%. To conclude, MCEplatelet value is related to both MCFplatelet and the platelet count.

To perform a sensitivity analysis, we divided the population into males and females, and we conducted separate analyses for the MCEplatelet relation to the MCFplatelet and platelet count. R^2^ was 61.0% and 56.8%, respectively, indicating that the model provides robust results separately for females and males and is consistent with the overall model (R^2^ = 56.7%). Gestational age was not a significant independent variable in both models (*p* = 0.6891 and 0.0852), while MCFplatelet and platelet count were significant. Regarding the females’ model, the estimates were 2.3 and 0.24, respectively (*p* = 0.0161 and <0.0001), and regarding the males’ model, the estimates were 4.69 and 0.20, respectively (*p* < 0.0001 in both cases). The latter findings are indicative of robust results.

### 3.6. MCFplatelet and MCEplatelet Correlations with Platelet Count and Platelet Function

We calculated all correlation coefficients separately for the sepsis and control groups to identify possible correlations for MCEplatelet and MCFplatelet with platelet count and flow cytometry characteristics (Supplementary Figure S1). Strong correlation coefficients were found for MCEplatelet with platelet count (PLTs) in the sepsis and control groups (r = 0.72, *p* < 0.0001 and r = 0.76, *p* < 0.001, respectively). Moderate correlation coefficients were found for MCEplatelet with GPIIb CD41receptors in the activated platelets of the control group (r = 0.35, *p* < 0.05). Strong correlation coefficients were found for MCFplatelet with platelets in the sepsis and control groups (r = 0.54, *p* < 0.0001 and r = 0.46, *p* < 0.01, respectively). Regarding flow cytometry, MCFplatelet was negatively correlated with GPIb CD42b MFI in inactivated platelets (r = −0.32, *p* = 0.003), with GPIb CD42b receptors in inactivated platelets (r = −0.31, *p* = 0.003) and GPIb CD42b MFI in activated platelets (r = −0.27, *p* = 0.01) in the sepsis group, and there were P-selectin 62p receptors in the inactivated platelets (r = −0.50, *p* = 0.004) in the control group.

### 3.7. MCFplatelet and MCEplatelet for Predicting Platelet Count and Platelet Function

**Figure 3:**
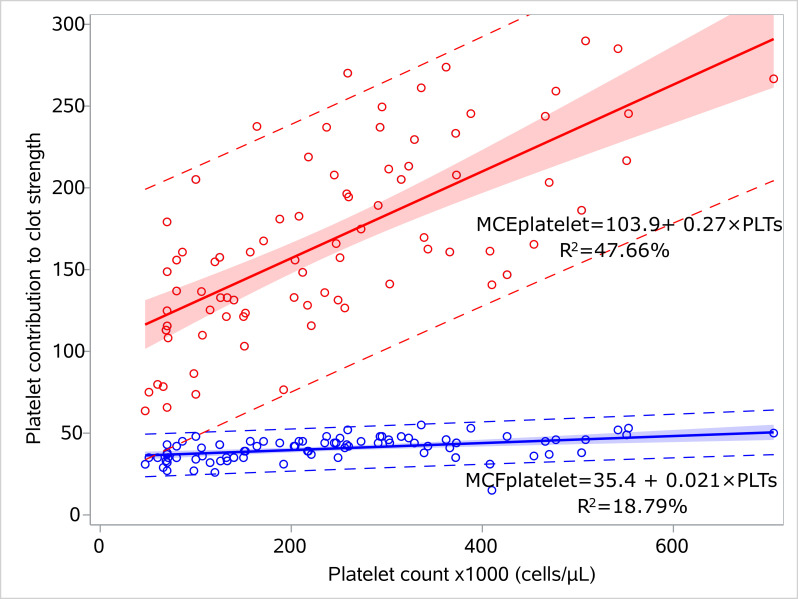
Linear models for platelet count and platelet contribution to clot strength for the septic neonates. Linear regression models visualizing the relation of MCFplatelet and MCEplatelet with platelet count for the sepsis group, including 95% prediction limits and 95% confidence interval. The circles indicate the actual measures. The linear equations and adjusted R2 values are depicted within the figures. Abbreviations: MCFplatelet: Maximum clot firmness for platelet; MCEplatelet: Maximum clot elasticity for platelet.

**Figure 4:**
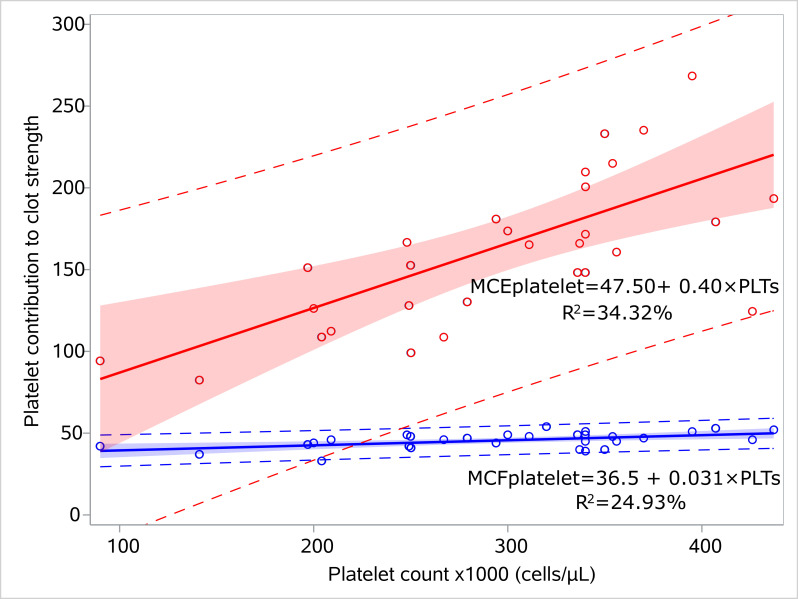
Linear models for platelet count and platelet contribution to clot strength for the controls. Linear regression models visualizing the relation of MCFplatelet and MCEplatelet with platelet count for the control group, including 95% prediction limits and 95% confidence interval. The circles indicate the actual measures. The linear equations and adjusted R2 values are depicted within the figures. Abbreviations: MCFplatelet: Maximum clot firmness for platelet; MCEplatelet: Maximum clot elasticity for platelet.

We performed regression analysis for MCEplatelet and platelet count, and similarly for MCFplatelet and platelet count for the sepsis (Figure 3, Supplementary Table S2) and control groups (Figure 4, Supplementary Table S3). MCEplatelet is a better estimator of platelet count than MCFplatelet since it has a better adjusted R^2^ in the sepsis (47.66% vs. 18.79%) and the control groups (34.32% vs. 24.93%), respectively.

We performed regression analysis for MCEplatelet and platelet flow cytometry, and similarly for MCFplatelet and platelet flow cytometry for patients and controls. MCEplatelet is poorly related to flow cytometry parameters (GPIIb, 0.36; GPIIIa, 0.32) despite P-selectin correlation coefficients being statistically significant in the control group. No relation was found between MCFplatelet and the flow cytometry parameters. Neither MCEplatelet nor MCFplatelet can predict flow cytometry results.

A multiple linear regression model was applied to investigate the relations of MCEplatelet and MCFplatelet with platelet count and GPIb CD42b receptors (both for activated and inactivated platelets). Both MCEplatelet and MCFplatelet have strong positive relations (*p* < 0.0001) with platelet count, meaning that increased platelet count results in increased MCEplatelet and MCFplatelet. No relation was confirmed for GPIb (Supplementary Table S4).

### 3.8. MCFplatelet and MCEplatelet Discrimination Capability to Detect Thrombocytopenia

**Figure 5:**
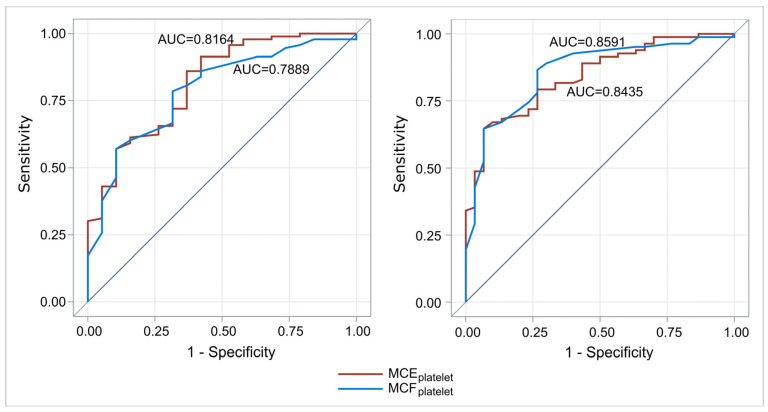
ROC curves for the discrimination capability of MCEplatelet and MCFplatelet to predict platelet count (left: limit 100,000/μL platelets; right: limit 150,000/μL platelets). For a limit of platelet count of <100,000/μL and <150,000 μL, MCEplatelet had better discrimination capability than MCFplatelet. Abbreviations: MCFplatelet: Maximum clot firmness for platelet; MCEplatelet: Maximum clot elasticity for platelet.

We evaluated the discrimination capability of MCEplatelet and MCFplatelet on platelet count status. Specifically, two analyses were performed for a platelet count limit of <100,000/μL and <150,000/μL, respectively. This analysis concerned the whole population (112 cases). For a platelet count limit of <100,000/μL, MCEplatelet had better discrimination capability than MCFplatelet (AUC = 0.82 and 0.79, respectively, *p* = 0.44). Similarly, for a platelet count limit of <150,000/μL, MCEplatelet has better discrimination capability than MCFplatelet (AUC = 0.86 and 0.84, respectively, *p* = 0.63) (Figure 5).

### 3.9. MCFplatelet and MCEplatelet for Reflecting Platelets and Fibrinogen Response

Linear regression analysis was applied to identify a possible relationship between EXTEM-MCF and FIBTEM-MCF with fibrinogen and platelet count, as well as with the sepsis group. The results are based on the aggregated data of all three study periods (Supplementary Table S5). Both for EXTEM and FIBTEM-MCF, increased fibrinogen or platelet count results in increased MCF; furthermore, an individual in the control group presents an increased risk for lower MCF values (*p* = 0.0613 for EXTEM-MCF and *p* < 0.0001 for FIBTEM-MCF). For EXTEM-MCF and MCFplatelet, an increase of 0.037 and 0.024 mm is recorded with an increase in platelet count of 10 × 10^3^/μL, and similarly, for MCEplatelet, an increase of 0.27 mm is expected (*p* < 0.00001), meaning that MCEplatelet better reflects a relevant increase in platelet count.

### 3.10. Platelet and Fibrinogen Contribution to Clot Strength

**Figure 6:**
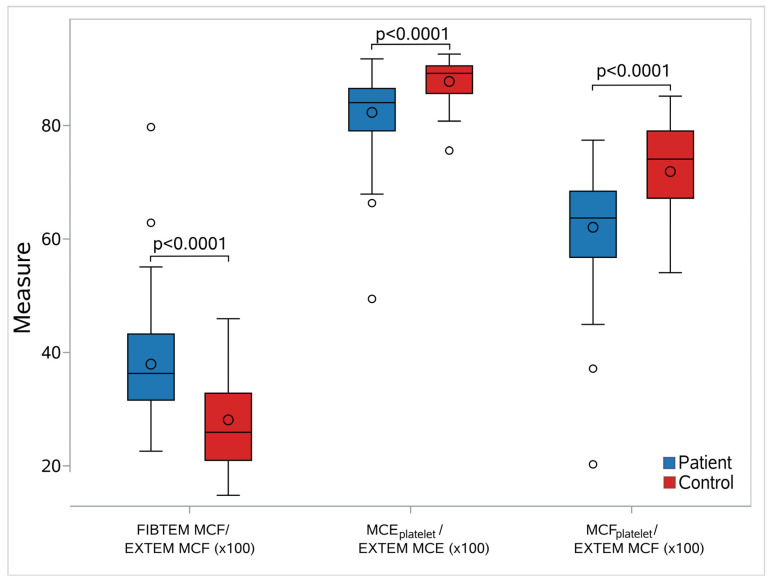
Box-whisker plots for the studied elasticity metrics between septic neonates and controls. In each diagram, the box limits indicate the lower (1st) and higher (3rd) quartiles (Q1 and Q3, respectively), the horizontal lines within the boxes indicate the median values, while the limits of the whiskers indicate the minimum and maximum values after excluding outliers. The mean values are visible as circles inside the boxes, while the circles outside the whisker areas indicate the outliers. For all cases, a significant difference in the elasticity values between septic neonates and controls was found (*p* < 0.0001). Abbreviations: FIBTEM: Fibrinogen rotational thromboelastometry; EXTEM: Extrinsic rotational thromboelastometry; MCF: Maximum clot firmness; MCFplatelet: Maximum clot firmness for platelet; MCEplatelet: Maximum clot elasticity for platelet.

We assessed the relative contribution of platelets and fibrinogen in the sepsis and the control groups. We calculated three additional quantities for the relative contribution of platelets and fibrinogen to clot formation: FIBTEM-MCF/EXTEM-MCFx100, MCEplatelet/EXTEM-MCEx100, and MCFplatelet/EXTEM-MCFx100. Then, we evaluated whether there was any difference in these indices between the sepsis and control groups. The relative contribution of fibrinogen was significantly higher (median 36.9 vs. 25.92, *p* < 0.001), and that of platelets was significantly lower (MCEplatelet median 84.03 vs. 89.21/MCFplatelet median 63.7 vs. 74.08; *p* < 0.001), in the sepsis group compared with the control group (Figure 6, Supplementary Table S6). For predicting platelet contribution to clot strength in patients via MCEplatelet and MCFplatelet, we used ROC curves. MCEplatelet (AUC 0.80) had better discrimination properties for the relative contribution of platelets in clot strength in the sepsis group than MCFplatelet (AUC 0.79), respectively.

### 3.11. Platelets and Fibrinogen Contribution to Clot Strength in EXTEM MCF Thresholds

Furthermore, we investigated the contribution of fibrinogen and platelet count as a discriminant factor for the participants who had an EXTEM-MCF of <55 mm or ≥55.

**Figure 7:**
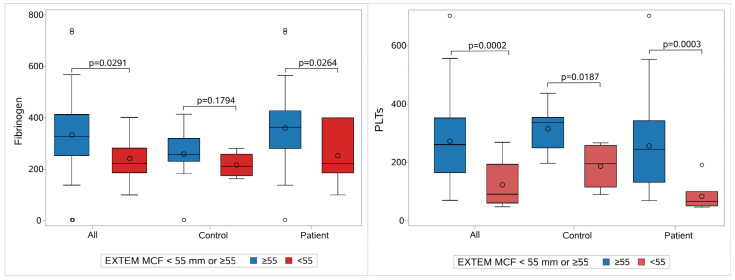
Fibrinogen and platelet contribution to clot strength. The contribution of the fibrinogen (on the left) and of platelets (on the right) to clot strength regarding the whole population, controls, and patients. Each group is divided into two subgroups: one with MCF Extem ≥ 55 mm (blue) and the other with MCF Extem < 55 mm (red). Abbreviations: EXTEM: Extrinsic rotational thromboelastometry; MCF: Maximum clot firmness; PLT: Platelet count.

This analysis was performed separately for the sepsis and control groups, as well as for the whole study population. For the complete population and when aggregating measurements from all days, we found 11 neonates with EXTEM-MCF <55 and 101 with ≥55. According to the results, fibrinogen is a good predictor (ROC AUC = 76.09%) of EXTEM-MCF status, with a cut-off value of 55. This was also confirmed separately for the sepsis group (*p* = 0.0264 and ROC AUC = 75.97%). However, it was not possible to prove this for the control group, as the number of neonates with EXTEM-MCF < 55 was rather limited (N = 4) (Figure 7, Supplementary Table S7).

Similarly, platelets proved to be an excellent predictor for EXTEM-MCF, with a cut-off value of 55 (ROC AUC = 84.52%, *p* < 0.0001 for comparison with the reference line) in the sepsis (ROC AUS = 91.90%, *p* < 0.0001) and control groups, respectively (ROC AUC = 87.02%, *p* < 0.0001).

## 4. Discussion

Our study showed that MCEplatelet is a better predictive index of platelet count among septic and healthy neonates and for the whole study population. Multivariate analysis revealed that MCEplatelet and MCFplatelet have strong positive relations with platelet count, meaning that increased platelet count results in increased MCEplatelet and MCFplatelet. The MCEplatelet has better discrimination capability than MCFplatelet for thrombocytopenia <100,000. MCEplatelet is poorly associated with platelet flow cytometry. However, neither MCEplatelet nor MCFplatelet can predict flow cytometry results among patients and their healthy counterparts. An increase in fibrinogen levels and clot strength in parallel with a decrease in platelet count was observed in neonates with sepsis compared with healthy neonates. Both fibrinogen and platelets contributed to clot strength, with fibrinogen being the main determinant of clot strength in preterm neonates with sepsis. Furthermore, a relevant increase in platelet count is better reflected by MCEplatelet. The contribution of platelets to MCF is lower compared to the fibrinogen contribution in neonates with sepsis. These results point to a tendency toward a hypercoagulable state attributed to fibrinogen due to the inflammatory response to sepsis, even in the presence of thrombocytopenia, suggesting that in neonatal sepsis, the simple difference between EXTEM and FIBTEM-MCF may not properly reflect the contribution of platelets to clot strength.

### 4.1. Elasticity-Based and Amplitude-Based Platelet Contribution to Clot Strength

Platelet contribution to the formation of a blood clot is reflected by the parameters of the performed tests that assess viscoelasticity with the use of the TEG or ROTEM technology, with or without platelet inhibition. In EXTEM, due to the extrinsic activation, initial thrombin generation and, hence, initial clotting mainly depend on the activity of the coagulation factors VII, X, V, and II and fibrinogen. Clot firmness in the EXTEM assay reflects both fibrin and platelet contributions to the strength of the clot. In FIBTEM, clot formation and clot strength depend only on fibrin formation and fibrin polymerization, as platelet function is inhibited [25]. Clot strength as well as clot amplitude can provide adequate information regarding the platelet element of the formed clot; however, platelet contribution toward clot strength has been suggested to more accurately indicate platelet deficiency than clot amplitude [9]. The resistance of blood clotting to the provided rotation is better estimated by clot elasticity, rather than clot amplitude, due to the non-linearity between the two parameters. In ROTEM, the measurement of clot amplitude reflects the range of resistance, while the thrombus process is advanced and derived from both the elasticity and viscosity properties of the forming clot. The clot amplitude ranges from 0 to 100 mm, and the maximal value is determined at random. Particularly, both viscosity and elasticity contribute to clot amplitude, but it is of doubtful validity to determine the contribution of each of these clot properties to clot amplitude using ROTEM or TEG, as clot amplitude follows a non-linear relationship with elasticity. Our data confirm this non-linearity hypothesis [9]. Particularly, when amplitude ranges in low values, our data depict that elasticity stands unrelated to amplitude. Moreover, these outcomes suggest not only that MCFplatelet and MCEplatelet are distinctly different variables but also that this discrepancy becomes more evident the greater the deviation in amplitude between the MCF-EXTEM and MCF-FIBTEM parameters. The latter is observed when the involvement of the platelets in clot strength is increased, but the MCF remains unchanged, suggesting that MCF in such cases is further independent. Indeed, our findings show that a low difference between MCFplatelet and MCEplatelet is recorded for low platelet contribution in severe thrombocytopenia. Conversely, for a platelet count of 100,000/μL, the difference between MCF and MCE is substantially larger. Additionally, our study results suggest that, when used to predict the severity of thrombocytopenia, MCE has a significantly higher discrimination capability than the MCF. Therefore, the assessment of platelet contribution to clot strength derived from ROTEM values to drive treatment protocols in bleeding should be under scrutiny before being approved in the neonatal population. Until recently, algorithms for the management of bleeding were structured based on MCF and were used by many researchers [26,27,28]. Thereafter, a strong indication is provided for the establishment of MCE instead of MCF to guide platelet transfusion policies in active bleeding [29]. Notwithstanding, the outcomes of our study are confirmatory of the recent literature in adults and still provide new perspectives on using such modalities in clinical settings in NICUs.

### 4.2. Elasticity-Based and Amplitude-Based Discrimination Capability to Predict Lower Platelet Count or Function

One of the primary outcomes in our study could be the assessment of the predictive value of MCFplatelet and MCEplatelet for both platelet count and function. Negative correlations between MCFplatelet and platelet function in subjects of the whole population are further indicative of the impairment of MCFplatelet as an index of platelet function. Most importantly, we could demonstrate that MCEplatelet is poorly correlated with platelet function. Conversely, MCEplatelet was strongly correlated with platelet count but still poorly associated with TRAP-dependent platelet function. The weak correlation between MCEplatelet and platelet function, as evaluated after triggering with TRAP, remains unclear. However, 80% of the ROTEM data that was coupled with platelet flow cytometry data came from very premature neonates with sepsis. Premature neonates below 32 weeks of GA exhibit platelet functional impairment, and neonatal platelets are presented as less reactive to natural or exogenous agonists [30,31]. Furthermore, during sepsis, thrombin is extensively formed, and platelets are consequently activated [13,14]. Therefore, this thrombin burst may partially impair the platelet response to TRAP, which is used to activate platelets in flow cytometry. The weakness in the correlation between MCEplatelet and platelet function may stem from the fact that the platelet component reflects poor platelet activation or function. However, the association between MCEplatelet and platelet function, as indicated by flow cytometry, should not be disregarded, even if the correlation appears weak. This finding may hold significant clinical relevance, and these results may have important clinical implications.

These observations are crucial for the decision-making process in transfusing bleeding patients. Currently, the platelet component of blood clot strength is not routinely used directly in treatment decisions. Platelet transfusions are typically guided by low whole blood clot strength (assessed by extrinsic activation) in the presence of adequate fibrin-based clot strength or even solely by a low MCF in EXTEM. Nuttal et al. suggested a platelet transfusion algorithm at an EXTEM-MCF cut-off limit of <48 mm with a platelet count < 102,000/μL [32]. Görlinger et al. suggested platelet transfusions in the setting of MCFplatelet < 30 mm at 10 min. Likewise, platelet transfusion was proposed by Karkouti et al. at MCFplatelet < 26 mm and thrombocytopenia < 75,000/μL. More recently, Sokou et al. suggested platelet transfusion in thrombocytopenic septic neonates with EXTEM-A10 < 37 mm as a strong predictor for bleeding events in this population [33]. The findings in our study challenge the current approach and advocate for an elasticity-based assessment as a more reliable indicator of the platelet component.

In our study, bleeding, along with severe thrombocytopenia, was recorded in only three patients. This does not support the recommendation of cut-off limits in ROTEM values, as the predictive value of bleeding suggests a need for platelet transfusion. However, our study showed that both fibrinogen and platelets contribute to EXTEM-MCF, and both have equally excellent predictive value for EXTEM-MCF < 55. We acknowledge that this value is specific to the institution and requires external validation before it is recommended. This finding implies that EXTEM-MCF cut-off limits should be carefully considered when guiding neonatal platelet transfusions. Moreover, the negative correlation between MCFplatelet and platelet function, the superior discrimination capability of MCEplatelet for platelet count, and the poor association of MCEplatelet with platelet function challenge the replacement of MCFplatelet by MCEplatelet in guiding transfusion policies in neonates.

In the future, the platelet component, calculated as the difference in clot elasticity between the whole blood clot and the fibrin-based clot, may be incorporated as a standard parameter in ROTEM analysis and validated against clinical outcomes. This advancement could enable the platelet component to serve directly as a quantitative index for guiding treatment decisions. Furthermore, there is a lack of clinical studies investigating the correlation between the ROTEM platelet component and platelet count and function. Viscoelastic tests utilize technology to assess coagulation under oscillatory conditions, eliminating the need for active blood flow. This setup simulates in vivo cases encountered during trauma and surgical procedures, where blood vessels are injured and transected, leading to interrupted blood flow and clot formation that seals the vessel (hemostatic clot). Neonates mostly suffer from sepsis, intrauterine or extrauterine hypoxia, or disseminated intravascular coagulation, all entities that (in vivo) present a variable hemostatic profile, which might be slightly different from that observed in trauma. Consequently, further research is warranted to elucidate the relationships between the platelet component, as assessed by ROTEM, and clinical morbidities in the neonatal population.

### 4.3. Contribution of Fibrinogen and Platelets to Clot Strength in Response to Sepsis

In our study, the relative fibrin contribution to clot strength was significantly higher, and the relative platelet contribution to maximum clot firmness was significantly lower in the septic neonates compared with their healthy counterparts. ROTEM allowed the identification of a tendency toward hypercoagulability in response to inflammation during sepsis. In our study, neonates with sepsis showed an increase in fibrinogen plasma levels and clot strength, pointing to a tendency toward a hypercoagulable state due to the inflammatory response to sepsis, even in the presence of severe thrombocytopenia. Fibrinogen and platelets contributed to clot strength, and fibrinogen could be the main determinant of blood hypercoagulability in these patients. The latter seems to be in line with the concept of immunothrombosis in patients with sepsis and may explain the lack of clear interpretation of the relationship between MCEplatelet and platelet function [34,35,36]. Levi et al. showed that a robust enhancement of clot strength and elasticity is observed in the initiation of sepsis [34]. Daudel et al. showed that a decrease in time of coagulation activation and clot formation, along with increased EXTEM-MCF, points to hypercoagulability in the early phase of sepsis [35]. Most importantly, studies showed that hypercoagulability was observed in ROTEM assays in the majority of patients with sepsis, except for those with overt Disseminated Intravascular Coagulation (DIC), who developed hypocoagulation and increased fibrinolysis [36]. The variations in coagulability in patients with sepsis that are recorded in the literature are consistent with the evolution of the disease over time [37]. Ostrowski et al. showed that fibrinogen was the main determinant of clot strength, independent of platelets, in both septic patients with hypocoagulability or hypercoagulability [38]. Hence, the results of our study indicate the need for a reassessment of platelet transfusion policy with a simultaneous consideration of fibrinogen contribution in sick preterm neonates with thrombocytopenia. Viscoelastic testing reduces the need for platelet transfusions and improves outcomes in critically ill patients with bleeding [39,40,41]. Speculation that the higher concentration of fibrinogen as an acute phase protein could compensate for the impairment of maximum clot firmness (MCF) caused by thrombocytopenia in sick neonates is of special interest.

The present study has limitations. The assessment of platelet function is limited, as we only used TRAP as an agonist. Researchers have employed various types of agonists and technologies to assess platelet activation. Assessing platelet function using different agonists in patients with various clinical conditions would be of clinical importance. Another important issue is the small sample size and the limited cases of clinical bleeding. Including a larger longitudinal cohort of patients with bleeding and conducting the study in multiple centers would enhance the robustness and validation of our results. This study has substantial strengths; it is a clinical study examining the trajectory of a disease, estimating ROTEM (a quantified method) and flow cytometry parameters (a qualitative method), and their association. This is the first study to encounter this entity in this type of population.

## 5. Conclusions

Our study provides evidence that MCEplatelet is a superior index for the platelet component of clot strength. From the perspective of routine clinical practice, MCEplatelet should be highly considered for guiding platelet transfusion in neonates at risk of bleeding. Equally, this study suggests evaluating whether supplementing fibrinogen concentrates in neonates with sepsis would improve the clot firmness compromised by severe thrombocytopenia. Thus, this study supports the potential to reduce platelet transfusions among preterm neonates. These findings are exploratory and primarily serve to generate hypotheses for further investigation. As such, they are not yet suitable for clinical application. Further research is required to clarify the implementation of such modalities in both preterm and term neonates. Particularly, to address the relationship between MCEplatelet, platelet function, bleeding, and the net role of fibrinogen in thrombocytopenic sick neonates, larger patient samples are required, and well-designed studies should be proposed.

## Figures and Tables

**Table 1:** Baseline characteristics of the study population. Patients are presented both as a single group and as Gram (+) and Gram (−) subgroups. *p*-values in bold indicate statistical significance at the level α = 0.05. Summary measures are expressed as “Median (IQR—Interquartile Range)” and as numbers with their respective percentages (%).

Characteristics	Confirmed Sepsis (N = 28)		*p*-Value Between Gram (+) and Gram (−)	All Patients (N = 28)	Controls (N = 30)	*p*-Value Between Patients and Controls
	Gram (+) (N = 25)	Gram (−) (N = 3)				
**General**						
GA (months)	31.7 (29–32.4)	27 (24.6–33.7)	0.44	31.4 (27.5–32.4)	31.4 (30.3–33)	0.37
BW (gr)	1400 (990–1735)	900 (740–2560)	0.44	1390 (970–1757.5)	1600 (1240–2080)	0.13
Gender (male)	13 (52%)	1 (33.3%)	0.54	14 (50%)	14 (46.7%)	>0.99
Mode of delivery (CS)	21 (84%)	3 (100%)	0.45	24 (85.7%)	28 (93.3%)	0.42
Caucasian race	All	All	>0.99	All	All	>0.99
IUGR	7 (28%)	1 (33.3%)	0.84	8 (28.6%)	6 (20%)	0.55
SGA	5 (20%)	0 (0%)	0.39	5 (17.9%)	3 (10%)	0.46
Survival	All	1 survivor	0.01	26 (92.9%) survived	All survived	0.23
PPROM	None	None		0 (0%)	2 (6.7%)	0.49
Bleeding observed	None	2 (66.7%)	0.01	2 (7.1%)	none	0.23
**Neonatal morbidities (any of the diseases below)**						
Morbidity (any from below)	19 (76%)	2 (66.7%)	0.72	24 (85.7%)	17 (56.7%)	**0.02**
RDS	19 (76%)	2 (66.7%)	0.72	21 (75%)	17 (56.7%)	0.17
IVH	1 (4%)	2 (66.7%)	0.02	3 (10.7%)	0 (0%)	>0.99
NEC	None	None	>0.99	None	None	>0.99
Microbiome	13 × Staphylococcus epidermidis 10 × Staphylococcus haemoliticus 2 × Staphylococcus hominis	2 × e-coli 1 × enterobacter		13 × Staphylococcus epidermidis 10 × Staphylococcus haemoliticus 2 × Staphylococcus hominis 2 × e-coli 1 × enterobacter	All negative	
**Laboratory tests**						
Thrombocytopenia (PLTs < 150 × 10^3^/μL)	8 (32%)	1 (33.3%)	0.96	9 (32.1%)	2 (6.7%)	**0.019**
PLT levels (×10^3^/μL)						
<50	0 (0%)	1 (33.3%)	0.14	1 (3.6%)	0 (0%)	0.06
50–100	5 (20%)	0 (0%)		5 (17.9%)	1 (3.3%)	
100–150	3 (12%)	0 (0%)		3 (10.7%)	1 (3.3%)	
>150	17 (68%)	2 (66.7%)		19 (67.9%)	28 (93.3%)	
CRP (mg/dL)	7.5 (3.5–8.2)	2.7 (2.5–8.8)	0.68	7.5 (2.6–8.4)	0.2 (0.1–0.2)	**<0.0001**
WBC (×10^6^/μL)	11,900 (9710–22,700)	16,000 (13,300–19,600)	0.66	14,650 (9855–21,800)	9955 (7170–12,300)	**0.027**
SGOT (U/L)	26 (22–43)	33 (21–51)	0.60	26.5 (21.5–43.5)	20.5 (18–30)	0.08
SGPT (U/L)	8 (6–22)	7 (5–9)	0.33	7.5 (6–21)	7 (6–11)	0.09
Total bilirubin (mg/dL)	7 (2.8–7.8)	5.5 (4.9–12.1)	0.71	6.4 (3.7–8)	7.3 (4.4–10)	0.12
Direct bilirubin (mg/dL)	0.5 (0.4–0.6)	0.4 (0.4–0.6)	0.41	0.5 (0.4–0.6)	0.5 (0.4–0.6)	0.37
Urea (mg/dL)	33 (22–43)	40 (25–42)	0.79	33 (23.5–43)	20.5 (14–34)	**0.004**
Creatinine (mg/dL)	0.5 (0.4–0.7)	0.6 (0.4–0.7)	0.82	0.5 (0.4–0.7)	0.6 (0.5–0.7)	0.77
**Maternal characteristics**						
Maternal age (years)	34 (30–40)	34 (30–45)	0.91	34 (30–40.5)	35 (31–38)	0.98
Disease (any from below)	15 (60%)	3 (100%)	0.53	18 (64.3%)	12 (40%)	0.07
Thyroid disease	7 (28%)	1 (33.3%)	0.85	8 (28.6%)	6 (20%)	0.45
Preeclampsia	3 (12%)	0 (0%)	0.53	3 (10.7%)	0 (0%)	0.11
HD	5 (20%)	1 (33.3%)	0.59	6 (21.4%)	2 (6.7%)	0.14
DM	4 (16%)	0 (0%)	0.45	4 (14.3%)	7 (23.3%)	0.38
Other disease (chorioamnionitis, coagulation issues)	1 (4%)	2 (66.7%) both chorioamnionitis	**0.02**	3 (10.7%)	2 (6.7%)	0.67
Medication (Yes)	12 (48%)	3 (100%)	0.23	15 (53.6%)	10 (33.3%)	0.19
Steroids	21 (84%)	2 (66.7%)	0.46	23 (82.1%)	27 (90%)	0.46

Abbreviations. GA: Gestational age; BW: Birth weight; CS: Caesarean section; IUGR: Intrauterine growth restriction; SGA: Small-for-gestational age; RDS: Respiratory distress syndrome; IVH: Intraventricular hemorrhage; NEC: Necrotizing enterocolitis; PPROM: Preterm premature rupture of the membranes; HD: Hypertensive disease; DM: Diabetes mellitus.

**Table 2:** ROTEM and flow cytometry values compared between patients and controls. Comparison was performed for each study period and for the aggregated data of all study patients. *p*-values in bold indicate statistical significance at the level α = 0.05.

		Control (N = 30) Median (IQR)	Day 1		Day 2–3		Day 5–7	
			Patients (N = 28) Median (IQR)	*p*	Patients (N = 28) Median (IQR)	*p*	Patients (N = 26 *) Median (IQR)	*p*
**Intem**	CT (s)	203 (172–250)	208 (178–243)	0.7371	214 (174–238)	1.0000	198 (165–210)	0.1414
	A10 (mm)	60 (56–66)	60 (56–65)	0.5218	63 (57–67)	0.6599	68 (64–71)	**0.0033**
	CFT (s)	61 (44–68)	70 (59–101)	**0.0361**	56.5 (45–76)	0.9344	42 (37–50)	**0.0028**
	MCF (mm)	64 (60–68)	63 (59–67)	0.5318	65 (62–70)	0.4199	71 (67–73)	**0.0023**
	LI30 (%)	98 (97–99)	98 (97–100)	0.7117	99 (98–100)	0.2670	98 (97–99)	0.7362
	LI45 (%)	95 (93–97)	95 (93–98)	0.4068	96 (95–97)	0.2261	95 (94–97)	0.5017
	LI60 (%)	91.5 (90–94)	92.5 (90–95)	0.2800	93 (92.5–95.5)	**0.0307**	93 (91.5–94)	0.1464
**Extem**	CT (s)	49.5 (45–55)	54 (49–61.5)	0.0863	51 (47–61.5)	0.3831	45.5 (43–51)	**0.0474**
	A10 (mm)	60 (55–66)	61.5 (55–66)	0.9193	62 (58–66.5)	0.4687	66.5 (59–72)	**0.0182**
	CFT (s)	67.5 (52–88)	73 (61–91.5)	0.0973	65.5 (48–81.5)	0.7438	54 (42–66)	**0.0271**
	MCF (mm)	65 (59–68)	64.5 (61–68.5)	0.9876	65 (61–71)	0.5589	70 (63–73)	**0.0152**
	LI30 (%)	99 (98–100)	99 (98–100)	0.6100	99.5 (98.5–100)	0.2300	99 (98–100)	0.7214
	LI45 (%)	95.5 (94–97)	96 (94–98)	0.3590	96.5 (94–98)	0.2232	95 (94–97)	0.9206
	LI60 (%)	93 (90–94)	93 (91–96)	0.2607	94 (92–96)	0.0611	93 (91.5–94.5)	0.4336
**Fibtem**	CT (s)	52 (47–58)	56 (47.5–60)	0.2099	49.5 (44–59.5)	0.5179	46 (43–49)	**0.0204**
	A10 (mm)	17 (12–24)	21 (17–25)	**0.0232**	22 (19.5–27)	**0.0042**	23 (20–30)	**0.0013**
	CFT (s)	165 (106–222)	162.5 (101–637)	0.6330	212 (104–363)	0.5682	111 (72–400)	0.3555
	MCF (mm)	17 (13–24)	23 (19.5–26.5)	**0.0037**	24 (21–29)	**0.0007**	25 (22–31)	**0.0002**
	LI30 (%)	100 (98–100)	100 (100–100)	**0.0273**	100 (100–100)	**0.0403**	100 (97–100)	0.3852
	LI45 (%)	99.5 (95–100)	100 (100–100)	**0.0125**	100 (99.5–100)	**0.0288**	100 (93–100)	0.7141
	LI60 (%)	99 (95–100)	100 (100–100)	**0.0155**	100 (100–100)	**0.0228**	100 (92–100)	0.6631
	MCE platelet	163 (126–193)	152 (127–180)	0.4184	157 (122–210)	0.8458	204 (147–238)	0.0525
	MCF platelet	46 (42–49)	42 (35–44)	**0.0004**	43 (35–45)	**0.0014**	42 (37–48)	0.0676
**Flow Cytometry**	GPIb CD42b MFI inactivated	2.5 (2.3–2.7)	2.8 (2.4–3)	0.0826	2.7 (2.5–2.9)	0.0648	2.8 (2.6–3.1)	**0.0027**
	GPIb CD42b inactivated receptors	7152 (6712–7917)	8152.5 (6888–8698)	0.0826	7770 (7211–8565)	0.0648	8035 (7476–9126)	**0.0027**
	GPIb 42b MFI activated	2.6 (2.3–2.7)	2.7 (2.5–3)	0.0507	2.6 (2.5–3)	0.0813	2.8 (2.6–3.1)	**0.0011**
	GPIb 42b activated receptors	7520 (6771–7917)	7946.5 (7152.5–8742)	**0.0498**	7681 (7152–8654)	0.0800	8314.5 (7564–9126)	**0.0011**
	GPIIb CD41 MFI inactivated	2.8 (2.6–3.1)	3 (2.8–3.3)	**0.0187**	3 (2.6–3.4)	0.1845	3.1 (2.9–3.4)	**0.0104**
	GPIIb CD41 inactivated receptors	8079 (7623–8979)	8845.5 (8226–9659)	**0.0191**	8890 (7564–10,014)	0.1846	8934.5 (8506–10,014)	**0.0106**
	GPIIb CD41 MFI activated	3.6 (3.1–3.8)	3.6 (3.3–3.9)	0.6020	3.4 (3–4.1)	0.7674	3.7 (3.3–4.1)	0.2780
	GPIIb CD41 activated receptors	10,474 (9126–11,262)	10,578 (9526–11,426)	0.6020	9925 (8801–12,126)	0.7613	10,920 (9570–12,130)	0.2708
	GPIIIa CD61 MFI inactivated	3.5 (3.3–4)	3.9 (3.6–4.3)	**0.0349**	3.8 (3.3–4.2)	0.3496	3.8 (3.6–4.2)	**0.0392**
	GPIIIa CD61 inactivated receptors	10,148 (9570–11,679)	11,366 (10,430–12,574)	**0.0349**	11,053 (9748–12,245)	0.3215	11,172 (10,578–12,454)	**0.0392**
	GPIIIa CD61 MFI activated	4.3 (4–4.7)	4.6 (4.1–4.8)	0.2397	4.2 (3.8–4.6)	0.7430	4.6 (4–5)	0.2207
	GPIIIa CD61 activated receptors	12,693 (11,589–13,891)	13,591 (12,006–14,041)	0.2460	12,454 (11,172–13,531)	0.7370	13,411 (11,589–14,702)	0.2366
	P-selectin CD62P MFI inactivated	1.2 (1.1–1.3)	0.9 (0.9–1.1)	**0.0005**	1 (0.9–1.1)	**0.0005**	0.9 (0.9–1)	**<0.0001**
	P-selectin CD62P inactivated receptors	3445 (3155–3710)	2774 (2748.5–3082)	**0.0006**	2787 (2737–3212)	**0.0004**	2762.5 (2720–2813)	**<0.0001**
	P-selectin CD62P MFI activated	1.2 (1.1–1.3)	1.2 (1.1–1.3)	0.8763	1.2 (1.1–1.2)	0.1951	1.2 (1.1–1.3)	0.4847
	P-selectin CD62P activated receptors	3503 (3328–3909)	3561 (3255.5–3923.5)	0.8701	3358 (3126–3764)	0.2698	3432.5 (3241–3619)	0.4645

Abbreviations. A10: Amplitude 10 min after clotting time; CFT, Clot formation time; CT: Clotting time; ROTEM: Rotational thromboelastometry; INTEM: Intrinsic rotational thromboelastometry; LI30, LI45, and LI60: Lysis index at 30, 45, and 60 min after clotting time; MCEplatelet: Platelet contribution to clot elasticity; MCFplatelet: Platelet contribution to clot firmness; EXTEM: Extrinsic rotational thromboelastometry; FIBTEM: Fibrinogen rotational thromboelastometry/rotational thromboelastometry assay for fibrin formation; Activated: Activated platelet state; Inactivated: Inactivated platelet state; MFI: Mean fluorescent intensity; GPIb: Platelet glycoprotein Ib; GPIIb: Platelet glycoprotein IIb; GPIIIa: Platelet glycoprotein IIIa. * 2 patients with early-onset sepsis died.

## Data Availability

The original contributions presented in the study are included in the article/Supplementary Material, further inquiries can be directed to the corresponding author.

## Supplementary Materials

The following supporting information can be downloaded at https://www.mdpi.com/article/10.3390/children12070948/s1: Figure S1: Color-coded correlation coefficients for MCEplatelet/MCFplatelet and D-dimers, platelet count and flow cytometry tests.; Table S1: Laboratory test results between patients and controls for each individual study period.; Table S2. Linear regression models for MCFplatelet and MCEplatelet with platelet count in sepsis group; Table S3. Linear regression models for MCFpaltelet and MCEplatelet with platelet count in control group; Table S4. Multivariable analysis of MCEplatelet and MCFplatelet with PLTs and GPIb CD42b; Table S5. Regression model for clot strength; Table S6. Contribution of fibrinogen and platelets to clot strength; Table S7. Contribution of fibrinogen and platelets and AUCs in cut-off points.

## Abbreviations

The following abbreviations are used in this manuscript:

**A10**	Amplitude at 10 min after CT (clotting time)
**aPTT**	Activated Partial Prothrombin Time
**AUC**	Area Under the Curve
**CCTs**	Conventional Coagulation Tests
**CFT**	Clot Formation Time
**CRP**	C-Reactive Protein
**CT**	Clotting Time
**DIC**	Disseminated Intravascular Coagulation
**EXTEM**	Extrinsic Rotational Thromboelastometry
**FIBTEM**	Fibrinogen Rotational Thromboelastometry (rotational thromboelastometry assay for fibrin formation)
**GA**	Gestational Age
**GP**	Glycoprotein
**INR**	International Normalized Ratio (INR)
**INTEM**	Intrinsic Rotational Thromboelastometry
**IQR**	Interquartile Range
**IVH**	Intraventricular Hemorrhage
**LI30, LI45, LI60**	Lysis Index at 30, 45, and 60 min after CT (clotting time)
**MCE**	Maximum Clot Elasticity
**MCEplatelet**	Platelet Contribution to Clot Elasticity
**MCF**	Maximum Clot Firmness
**MCFplatelet**	Platelet Contribution to Clot Firmness
**MFI**	Mean Fluorescence Intensity
**NICU**	Neonatal Intensive Care Unit
**PLTs**	Platelet Count
**PRP**	Platelet Rich Plasma
**PT**	Prothrombin Time
**RIA**	Radioimmunoassay
**ROTEM**	Rotational Thromboelastometry
**SD**	Standard Deviation
**SNAPPE II**	Score for Neonatal Acute Physiology Perinatal Extension II
**TRAP**	Thrombin Receptor Activating Peptide
**WBC**	White Blood Cells

## Appendix A

- *Glossary*
- EXTEM: Extrinsic rotational thromboelastometry. In the EXTEM assay, clot formation is induced by the activation of the extrinsic coagulation pathway using 20 μL of 0.2 mol/L calcium chloride solution (star-TEM reagent) and 20 μL of extrinsic activator (ex-TEM reagent; recombinant tissue factor and phospholipids).
- FIBTEM: Fibrinogen rotational thromboelastometry (rotational thromboelastometry assay for fibrin formation). The FIBTEM assay constitutes a modified EXTEM assay, where platelet contribution to clot formation is inhibited with 20 μL of thrombocyte inhibitor (fib-TEM reagent; Cytochalasin D and 0.2 mol/L of calcium chloride).
- MCF: Maximum clot firmness. MCF is calculated separately for EXTEM and FIBTEM. MCF reflects the final strength of the clot.
- MCE: Maximum clot elasticity. MCE is calculated as MCE = (100 × MCF)/(100 − MCF) (separately for EXTEM and FIBTEM). MCE describes the mechanical properties of the clot.
- MCFplatelet: Platelet contribution to clot firmness. MCFplatelet = (MCF-EXTEM) − (MCF-FIBTEM).
- MCΕplatelet: Platelet contribution to clot elasticity. MCEplatelet = (MCE-EXTEM) − (MCE-FIBTEM).
