# Supplementary material for: Platelet and Fibrinogen Contribution to Clot Strength in Premature Neonates with Sepsis
Source: Children (Basel). 2025 Jul 18;12(7):948. doi: 10.3390/children12070948 (PMC12293396; doi:10.3390/children12070948)
Supplement: Supplementary file 1 [file children-12-00948-s001.zip › children-3720724-supplementary.pdf]

# SUPPLEMENTARY TABLES

*Table S1.* Laboratory test results between patients and controls for each individual study period. Statistically significant P-values at level α=0.05 are given in bold.

| | Control (N=30) Median (IQR) | Day 1 | | Day 2-3 | | Day 5-7 | |
|---|---|---|---|---|---|---|---|
| | | Patients (N=28) Median (IQR) | p | Patients (N=28) Median (IQR) | p | Patients (N=28) Median (IQR) | p |
| **PT (sec)** | 12.6 (11.9-13.3) | 14.4 (13.2-17.6) | **<0.0001** | 13 (12.3-14.95) | **0.0438** | 12.65 (12.3-13.3) | 0.5108 |
| **INR** | 0.95 (0.91-1.03) | 1.095 (1-1.34) | **<0.0001** | 0.95 (0.925-1.11) | 0.2821 | 0.96 (0.91-1) | 0.9672 |
| **APTT (sec)** | 30.15 (26.4-33.7) | 39.05 (36-47.45) | **<0.0001** | 35.45 (31.5-42.55) | **0.0017** | 35.1 (32.1-36.8) | **0.0051** |
| **Fibrinogen (mg/dL)** | 253 (219-316) | 400 (319-464) | **<0.0001** | 384.5 (276-439.5) | **0.0005** | 293.5 (216-379) | 0.1328 |
| **D-Dimers (ng/mL)** | 1175 (790-1475) | 2095 (1660-3600) | **<0.0001** | 2210 (1900-3540) | **<0.0001** | 1635 (1100-2280) | **0.0339** |
| **Protein C (%)** | 31.5 (28-38) | 22 (18-26) | **<0.0001** | 26.5 (21-33) | 0.1758 | 31 (28-35) | 0.9357 |
| **Protein S (%)** | 50 (45-57) | 47 (36-59) | 0.2555 | 48 (35-56) | 0.5943 | 49 (43-59) | 0.8511 |
| **vWF (U)** | 172 (155-209) | 221 (200-306) | **0.0026** | 200 (171-218) | 0.4112 | 206 (164-224) | 0.4376 |
| **WBC (x10$^6$/μL)** | 9955 (7170-12300) | 14650 (9855-21800) | **0.0266** | 15700 (9965-18700) | **0.0098** | 13950 (9350-16100) | **0.0090** |
| **Hemoglobin (g/dL)** | 14 (12.8-16.2) | 12.45 (10.15-14.3) | **0.0152** | 11.5 (10.5-12.85) | **0.0002** | 10.8 (8.9-11.7) | **<0.0001** |
| **Hematocrit (%)** | 41 (37-46.8) | 36.9 (29.6-39.95) | **0.0299** | 33.65 (30.7-38.5) | **0.0004** | 31.3 (26-34) | **<0.0001** |
| **PLT (x10$^3$/μL)** | 315.5 (249-350) | 208 (119-258.5) | **0.0002** | 187 (99-313) | **0.0051** | 340.5 (140-477) | 0.6513 |
| **PLT FC (x10$^3$/μL)** | 281 (200-375) | 89 (57-155) | **<0.0001** | 106.5 (50-157) | **<0.0001** | 165 (56-240) | **0.0021** |
| **Reticulocytes (%)** | 3.45 (1.8-5.1) | 2.9 (1.5-4) | 0.5539 | 1.95 (1.3-2.6) | **0.0293** | 1.8 (1.6-2.3) | **0.0249** |
| **Neutrophils** | 3335 (2270-4370) | 8295 (3800-11850) | **0.0006** | 5360 (2730-9755) | **0.0282** | 4645 (2540-7100) | 0.1371 |
| **CRP (mg/dL)** | 0.165 (0.13-0.24) | 7.46 (2.595-8.445) | **<0.0001** | 2.86 (1.21-6.405) | **<0.0001** | 0.48 (0.21-0.82) | **0.0011** |
| **Urea (mg/dL)** | 20.5 (14-34) | 33 (23.5-43) | **0.0036** | 30 (20.5-38.5) | **0.0406** | 17.5 (12-25) | 0.3702 |
| **Creatinine (mg/dL)** | 0.6 (0.5-0.7) | 0.545 (0.44-0.73) | 0.7673 | 0.54 (0.455-0.67) | 0.3582 | 0.5 (0.42-0.57) | **0.0200** |
| **SGOT (U/L)** | 20.5 (18-30) | 26.5 (21.5-43.5) | 0.0782 | 22.5 (15.5-36) | 0.9627 | 18.5 (15-26) | 0.1728 |
| **SGPT (U/L)** | 7 (6-11) | 7.5 (6-21) | 0.0857 | 10 (6.5-19) | **0.0117** | 10 (7-15) | **0.0021** |
| **Total bilirubin (mg/dL)** | 7.285 (4.35-10) | 6.375 (3.71-7.995) | 0.1523 | 6.24 (4.305-8.12) | 0.0899 | 3.865 (2-5.27) | **0.0001** |
| **Direct bilirubin (mg/dL)** | 0.49 (0.43-0.57) | 0.495 (0.42-0.635) | 0.3663 | 0.6 (0.46-0.73) | **0.0148** | 0.485 (0.41-0.66) | 0.3572 |

Abbreviations. PT, prothrombin time; APTT, activated partial thromboplastin time; vWF, Von Willebrand factor; WBC; White Blood Cells; PLT, platelets; Platelet FC, platelets estimated by flow cytometry; CRP, C-reactive protein; SGOT, serum glutamic oxaloacetic transaminase; SGPT, glutamate-pyruvate transaminase

*Table S2*. Linear regression models for MCFplatelet and MCEplatelet with platelet count in sepsis group.

| $\mathbf{MCF_{platelet}}$ **and Platelets** | $\mathbf{MCE_{platelet}}$ **and Platelets** |
|---|---|
| Intercept = 35.4, Standard Error= 1.4, $p<0.0001$ and β=0.021, Standard Error=0.0048, $p<0.0001$ | Intercept = 103.9, Standard Error= 8.68, $p<0.0001$ and β=0.27, Standard Error=0.03, $p<0.0001$ |
| the linear model produced by this regression analysis is: $MCF_{platelet}$=35.4 + 0.021*PLTs, with overall $p<0.0001$ and adjusted **$R^2$=18.79%.** | the linear model produced by this regression analysis is: $MCE_{platelet}$=103.9+ 0.27*PLTs, with overall $p<0.0001$ and adjusted **$R^2$=47.66%.** |
| Abbreviations. MCE, platelet contribution to clot elasticity; MCFplatelet, platelet contribution to clot firmness | |

*Table S3*. Linear regression models for MCFpaltelet and MCEplatelet with platelet count in control group.

| $\mathbf{MCF_{platelet}}$ **and Platelets** | $\mathbf{MCE_{platelet}}$ **and Platelets** |
|---|---|
| Intercept = 36.5, Standard Error= 2.94, $p<0.0001$ and β=0.031, Standard Error=0.0095, $p=0.0029$ | Intercept = 47.50, Standard Error= 30.36, $p<0.1290$ and β=0.40, Standard Error=0.10, $p=0.0004$ |
| the linear model produced by this regression analysis is: $MCF_{platelet}$=36.5 + 0.031*PLTs, with overall $p<0.0029$ and adjusted **$R^2$=24.93%.** | the linear model produced by this regression analysis is: $MCE_{platelet}$=47.50+ 0.40*PLTs, with overall $p<0.0004$ and adjusted **$R^2$=34.32%.** |
| Abbreviations. MCE, platelet contribution to clot elasticity; MCFplatelet, platelet contribution to clot firmness | |

*Table S4.* Multivariable analysis of MCEplatelet and MCFplatelet with PLTs and GPIb CD42b.

| | MCEplatelet | | | MCFplatelet | | |
|---|---|---|---|---|---|---|
| **Variable** | **Estimate** | **Standard Error** | **p** | **Estimate** | **Standard Error** | **p** |
| **Platelets** | 0.27626072 | 0.03101817 | <.0001 | 0.0211723 | 0.0043699 | <.0001 |
| **GPIb CD42b active** | 20.5581237 | 18.4749733 | 0.2683 | -1.9417161 | 2.6027913 | 0.4573 |
| **GPIb CD42b inactive** | -8.10139634 | 19.4653216 | 0.6781 | -1.9215458 | 2.7423135 | 0.485 |

Abbreviations. MCE, platelet contribution to clot elasticity; MCFplatelet, platelet contribution to clot firmness

*Table S5.* Regression model for clot strength.

| | | **Coefficient** | **Standard Error** | **p** |
|---|---|---|---|---|
| **Extem MCF** | Intercept | 51.7 | 1.92 | <0.0001 |
| | Fibrinogen | 0.014 | 0.004 | 0.0016 |
| | PLTs count | 0.037 | 0.004 | <0.0001 |
| | Control | -2.37 | 1.25 | 0.0613 |
| | Patients (reference) | | | |
| **Fibtem MCF** | Intercept | 21.3 | 2.04 | <0.0001 |
| | Fibrinogen | 0.011 | 0.005 | 0.0564 |
| | Control | -5.7 | 1.5 | 0.0004 |
| | Patients (reference) | | | |
| **MCFplatelet** | Intercept | 35.6 | 1.24 | <0.0001 |
| | PLTs count | 0.024 | 0.004 | <0.0001 |
| **MCEplatelet** | Intercept | 98.7 | 8.6 | <0.0001 |
| | PLTs count | 0.27 | 0.03 | <0.0001 |

Abbreviations. EXTEM: extrinsic coagulation pathway; FIBTEM: extrinsic coagulation pathway with platelet inhibition by cytochalasin D; MCF: maximum clot firmness; MCFplatelet: platelet contribution to clot firmness; MCEplatelet: platelet contribution to clot elasticity; Intercept: value at which the adjusted line crosses the y-axis

*Table S6*. Contribution of fibrinogen and platelets to clot strength.

| Characteristic | Patients (N=82) | | Controls (N=30) | | p |
|---|---|---|---|---|---|
| | Median | Q1-Q3 | Median | Q1-Q3 | |
| **FIBTEM MCF/EXTEM MCF x 100** | 36.30 | (31.58-43.24) | 25.92 | (20.97-32.81) | <0.0001 |
| **MCEplatelet/EXTEM MCE x 100** | 84.03 | (79.06-86.54) | 89.21 | (85.64-90.52) | <0.0001 |
| **MCFplatelet/EXTEM MCF x 100** | 63.70 | (56.76-68.42) | 74.08 | (67.19-79.03) | <0.0001 |

Abbreviations. EXTEM: extrinsic coagulation pathway; FIBTEM: extrinsic coagulation pathway with platelet inhibition by cytochalasin D; MCF: maximum clot firmness; MCEplatelet: platelet contribution to clot elasticity; MCFplatelet: platelet contribution to clot firmness

*Table S7*. Contribution of fibrinogen and platelets and AUCs in cut-off points.

| | EXTEM MCF | Complete population | | | Patients | | | Controls | | |
|---|---|---|---|---|---|---|---|---|---|---|
| | | N | Median (Q1-Q3) | p | N | Median (Q1-Q3) | p | N | Median (Q1-Q3) | p |
| **Fibrinogen** | <55 | 11 | 186 (281-95) | **0.0291** | 7 | 222 (186-400) | **0.0264** | 4 | 211.5 (175-258) | 0.1794 |
| | ≥55 | 101 | 326 (251-411) | | 75 | 363 (281-427) | | 26 | 257.5 (231-320) | |
| | **EXTEM MCF** | **Complete population** | | | **Patients** | | | **Controls** | | |
| | | **N** | **Median (Q1-Q3)** | **p** | **N** | **Median (Q1-Q3)** | **p** | **N** | **Median (Q1-Q3)** | **p** |
| **PLTs count** | <55 | 11 | 90 (60-192) | **0.0002** | 7 | 66 (51-100) | **0.0003** | 4 | 195.5 (115.5-258.5) | **0.0187** |
| | ≥55 | 101 | 259 (164-350) | | 75 | 245 (132-343) | | 26 | 336.5 (250-354) | |

Abbreviations. EXTEM: extrinsic coagulation pathway; MCF: maximum clot firmness; PLTs: platelets

# SUPPLEMENTARY FIGURES

| | Patients | Patients | Control | Control | All | All |
|---|---|---|---|---|---|---|
| | MCE platelet | MCF platelet | MCE platelet | MCF platelet | MCE platelet | MCF platelet |
| D-dimers (ng/mL) | -0.29* | -0.37* | -0.23 | 0.04 | -0.21* | -0.4*** |
| Platelets ($x10^3/\mu L$) | 0.72*** | 0.54*** | 0.66*** | 0.46* | 0.66*** | 0.55*** |
| Platelet FC ($x10^3/\mu L$) | 0.65*** | 0.58*** | 0.76* | 0.21 | 0.54*** | 0.59*** |
| GPIb CD42b MFI inactivated | -0.13 | -0.32* | 0.11 | -0.1 | -0.06 | -0.34* |
| GPIb CD42b inactivated receptors | -0.13 | -0.32* | 0.11 | -0.1 | -0.06 | -0.34* |
| GPIb CD42b MFI activated | -0.06 | -0.27* | 0.2 | -0.14 | 0.02 | -0.29* |
| GPIb CD42b activated receptors | -0.06 | -0.27* | 0.2 | -0.14 | 0.02 | -0.29* |
| GPIIb CD41 MFI inactivated | 0.02 | -0.17 | 0.23 | 0.01 | 0.08 | -0.19* |
| GPIIb CD41 inactivated receptors | 0.02 | -0.17 | 0.23 | 0.01 | 0.08 | -0.19* |
| GPIIb CD41 MFI activated | 0.08 | -0.11 | 0.36 | 0.17 | 0.15 | -0.08 |
| GPIIb CD41 activated receptors | 0.08 | -0.11 | 0.36 | 0.17 | 0.15 | -0.08 |
| GPIIIa CD61 MFI inactivated | -0.03 | -0.18 | 0.1 | -0.03 | 0.01 | -0.22* |
| GPIIIa CD61 inactivated receptors | -0.04 | -0.18 | 0.1 | -0.03 | 0.01 | -0.22* |
| GPIIIa CD61 MFI activated | -0.02 | -0.2 | 0.32 | 0.08 | 0.06 | -0.16 |
| GPIIIa CD61 activated receptors | -0.02 | -0.2 | 0.32 | 0.08 | 0.06 | -0.16 |
| P-selectin CD62P MFI inactivated | -0.09 | -0.11 | -0.22 | -0.51* | -0.13 | -0.01 |
| P-selectin CD62P inactivated receptors | -0.06 | -0.09 | -0.22 | -0.51* | -0.11 | 0.01 |
| P-selectin CD62P MFI activated | 0.16 | 0.14 | 0 | 0.08 | 0.13 | 0.15 |
| P-selectin CD62P activated receptors | 0.15 | 0.14 | 0 | 0.08 | 0.12 | 0.14 |

+1

-1

*Figure S1*. Color-coded correlation coefficients for MCEplatelet/MCFplatelet and D-dimers, platelet count and flow cytometry tests. The analysis was performed for patients, controls and for the whole population. In each cell, the spearman correlation coefficient's value is represented by its colour and intensity. P-values are depicted as *:<0.05, **<0.001, ***<0.0001.
